# Ketolytic and glycolytic enzymatic expression profiles in malignant gliomas: implication for ketogenic diet therapy

**DOI:** 10.1186/1743-7075-10-47

**Published:** 2013-07-05

**Authors:** Howard T Chang, Lawrence Karl Olson, Kenneth A Schwartz

**Affiliations:** 1 Department of Neurology and Ophthalmology, Michigan State University, East Lansing, MI, 48824, USA; 2 Department of Pathology, Sparrow Hospital, Lansing, MI, 48912, USA; 3 Department of Physiology, Michigan State University, East Lansing, MI, 48824, USA; 4 Department of Medicine, Michigan State University, East Lansing, MI, 48824, USA

**Keywords:** OXCT1: Succinyl CoA: 3-oxoacid CoA transferase 1 (SCOT EC 2.8.3.5 locus symbol OXCT), BDH1: 3-hydroxybutyrate dehydrogenase 1, BDH2: 3-hydroxybutyrate dehydrogenase 2, ACAT1: acetyl-CoA acetyltransferase 1, HK2: Hexokinase-II, PKM2: Pyruvate kinase M2 isoform

## Abstract

**Background:**

Recent studies in animal models, based on the hypothesis that malignant glioma cells are more dependent on glycolysis for energy generation, have shown promising results using ketogenic diet (KD) therapy as an alternative treatment strategy for malignant glioma, effectively starving glioma cells while providing ketone bodies as an energy source for normal neurons and glial cells. In order to test this treatment strategy in humans, we investigated the relative expression of several key enzymes involved in ketolytic and glycolytic metabolism in human anaplastic glioma (WHO grade III) and glioblastoma (GBM, WHO grade IV).

**Methods:**

Immunohistochemistry was performed on formalin fixed paraffin embedded sections from 22 brain biopsies (17 GBM, 3 anaplastic astrocytoma and 2 anaplastic oligoastrocytoma) using antibodies raised against glycolytic and ketolytic enzymes. The glycolytic enzymes included hexokinase-II (HK2) and pyruvate kinase M2 isoform (PKM2). The ketone body metabolic enzymes included: succinyl CoA: 3-oxoacid CoA transferase (OXCT1), 3-hydroxybutyrate dehydrogenase 1 and 2 (BDH1 and BDH2), and acetyl-CoA acetyltransferase 1 (ACAT1). The immunoreactivities were graded using a semi-quantitative scale based on the percentage of positive cells: POS (>20%), LOW (5-20%), and very low (VLOW) (<5%). Focal non-neoplastic “normal” brain tissue within the biopsy specimens served as internal controls.

**Results:**

The rate limiting mitochondrial ketolytic enzymes (OXCT1 and BDH1) were either LOW or VLOW, concordantly in 14 of the 17 GBMs and in 1 of 5 anaplastic gliomas, whereas at least one of the glycolytic enzymes was POS in 13 of these 17 GBMs and all 5 anaplastic gliomas. Cytosolic BDH2 and mitochondrial ACTAT1 were, surprisingly, POS in most of these tumors.

**Conclusion:**

Our results showing that malignant gliomas have differential expression of ketolytic and glycolytic enzymes are consistent with previous studies that have shown that these are genetically heterogeneous tumors. It seems reasonable to hypothesize that patients with low or very low expression of key ketolytic enzymes in their malignant gliomas may respond better to the KD therapy than those patients with positive expression of these enzymes. Further studies in animal models and/or a large-scale clinical trial would be needed to test this hypothesis.

## Introduction/Background

Malignant gliomas including grade III (anaplastic) astrocytoma and grade IV astrocytoma (also known as glioblastoma, GBM) are among the leading causes of death from solid tumors in children and adults. Median survival with current standard treatments is between 12 and 18 months, and experimental therapies do not appear to be very effective, possibly due to genetic instability and heterogeneity of these tumors [1-3]. A promising novel approach has been demonstrated recently in rodents with orthotopically transplanted malignant glioma cells such that these rodents showed increased survival when fed a ketogenic diet (KD) [4,5]. Recent studies in rodents also showed that glioma tumor cells are more dependent on glycolysis for energy generation [6], and that KD reduced reactive oxygen species production in tumor cells [7]. Despite several case reports of KD therapy in human glioma patients [8,9], questions remained on whether KD may be applied effectively in humans. In order to better understand the metabolism of ketone bodies in human gliomas, we investigated the expression of several key enzymes involved in glucose and ketone body metabolism, using immunohistochemistry with specific antibodies, in human anaplastic glioma (WHO grade III) and glioblastoma (GBM, WHO grade IV) samples. Our results suggest that the differential expression of these enzymes could serve as potentially useful biomarkers to select human glioma patients who may or may not respond optimally to KD.

## Materials and methods

Immunohistochemistry reactions were performed on formalin fixed paraffin embedded sections from brain biopsies, using antibodies raised against several glycolytic and ketolytic enzymes.

### Glycolytic enzymes

Hexokinase-II (HK2): Hexokinases catalyze the essentially irreversible first step of the glycolytic pathway where glucose is phosphorylated to glucose-6-phosphate via phosphate transfer from ATP. Hexokinase-II (HK2) is bound to the outer membrane of mitochondria and constitutes the principal isoform in many cell types, and is increased in many cancers [10,11].

Pyruvate kinase M2 isoform (PKM2), an alternatively spliced variant of pyruvate kinase, is a cytosolic glycolytic enzyme that catalyses the conversion of phosphoenolpyruvate to pyruvate, and has been shown to be essential for aerobic glycolysis in many tumors [12].

### Ketone body metabolic enzymes

Succinyl CoA: 3-oxoacid CoA transferase 1 (OXCT1), encoded by the OXCT1 gene in human, is a mitochondrial enzyme that catalyzes the transfer of coenzyme A from succinyl-coenzyme A to acetoacetate, forming acetoacetyl-CoA, and is the key enzyme of ketone body utilization [6,13,14].

D-beta-hydroxybutyrate dehydrogenase (BDH1), encoded by the BDH1 gene in human, is a mitochondrial enzyme that catalyzes the interconversion of acetoacetate and (R)-3-hydroxybutyrate, the two major ketone bodies produced during fatty acid catabolism [6].

BDH2 is a cytosolic type 2 (R)-hydroxybutyrate dehydrogenase, distinct from the mitochondrial BDH1, may have role in cytosolic ketone body utilization, either as a secondary system for energy supply in starvation or to generate precursors for lipid and sterol synthesis [15].

Acetyl-CoA acetyltransferase (ACAT1), also known as acetoacetyl-CoA thiolase, encoded by the ACAT1 gene in human, is a mitochondrial enzyme that catalyzes the reversible formation of acetoacetyl-CoA from two molecules of acetyl-CoA [6].

### Specimens

Archival de-identified formalin fixed paraffin embedded brain biopsy specimens were selected for this study. Preferences were given whenever possible to select blocks that contain both malignant tumor and small portions of adjacent relatively normal brain tissue that would serve as internal normal control for the immunohistochemistry reactions. These included 17 glioblastomas (GBM), 3 anaplastic astrocytomas (AA), and 2 anaplastic oligodendrogliomas (AO) (Table 1).

**Table 1:** List of patients, and summary of the immunohistochemical reaction results in this study

	**Age/Gender**	**Diagnosis**	**GFAP**	**OXCT1**	**BDH1**	**BDH2**	**ACAT1**	**HK2**	**PKM2**
1	66M	GBM	POS	VLOW	VLOW	POS	POS	POS	POS
2	62M	GBM	POS	VLOW	VLOW	POS	POS	POS	POS
3	67F	GBM	POS	VLOW	VLOW	POS	LOW	LOW	POS
4	78M	GBM	LOW	VLOW	VLOW	LOW	VLOW	LOW	POS
5	45M	GBM	POS	VLOW	VLOW	POS	POS	VLOW	POS
6	43M	GBM/GS	VLOW	VLOW	LOW	POS	POS	POS	POS
7	55M	GBM	POS	VLOW	VLOW	POS	POS	VLOW	POS
8	35F	GBM	LOW	VLOW	VLOW	POS	LOW	VLOW	LOW
9	38M	GBM	LOW	POS	LOW	POS	POS	LOW	POS
10	69F	GBM	LOW	POS	LOW	POS	POS	LOW	LOW
11	35M	GBM	LOW	POS	LOW	POS	POS	VLOW	POS
12	45M	GBM/GS	POS	LOW	LOW	POS	POS	LOW	POS
13	85M	GBM	LOW	VLOW	VLOW	POS	LOW	POS	POS
14	66M	GBM	POS	LOW	LOW	POS	POS	POS	POS
15	30M	GBM	LOW	VLOW	VLOW	VLOW	POS	VLOW	VLOW
16	41M	GBM	LOW	LOW	VLOW	LOW	POS	VLOW	VLOW
17	78M	GBM	POS	LOW	VLOW	POS	LOW	LOW	POS
18	39M	AA	LOW	POS	LOW	POS	POS	VLOW	POS
19	70F	AA	POS	POS	POS	POS	POS	LOW	POS
20	71M	AA	LOW	LOW	LOW	POS	POS	POS	POS
21	40F	AOA	POS	POS	VLOW	POS	POS	VLOW	POS
22	62M	AOA	POS	POS	LOW	POS	POS	LOW	POS

#### Immunohistochemistry procedures

Formalin-fixed, paraffin-embedded tissue sections (5 μm thick) mounted on glass slides were deparaffinized, rehydrated, and underwent heat induced epitope retrieval utilizing citrate buffer (pH 6.0) for 30 minutes at 100°C. The slides were rinsed in water, and then immersed in 3% hydrogen peroxide/methanol bath for 30 minutes to block endogenous peroxidase. Following these pretreatments, the slides were subjected to standard avidin-biotin complex immunohistochemistry staining reactions performed at room temperature in a Dako Autostainer utilizing two, two-minute rinses between each staining steps. The sections were incubated in the primary antibodies for 60 minutes, followed by appropriate biotinylated secondary antibodies for 30 minutes, the Vectastain Elite ABC Reagent (Vector) for 30 minutes, and then developed using Nova Red (Vector) Peroxidase substrate kit for 15 minutes. The slides were rinsed in distilled water, counterstained with hematoxylin, rinsed, dehydrated through ascending grades of ethanol, cleared through xylene, and coverslipped using Flotex permanent mounting media.

The primary antibodies used in this study were: rabbit polyclonal antibody to glial fibrillary acidic protein (GFAP, Dako Z0334, Carpinteria, CA), mouse monoclonal antibody to hexokinase II (HK2, Abcam ab104836, Cambridge, MA), rabbit monoclonal antibody to human pyruvate kinase M2 isoform (PKM2, Cell Signaling Technology D78A4, Danvers, MA), rabbit polyclonal antibody to OXCT1 (3-oxoacid-CoA transferase 1, Sigma HPA012047, St. Louis, MO), mouse monoclonal antibody to mitochondrial beta-hydroxybutyrate dehydrogenase (BDH1, clone 1A5, ProMab 30003, Richmond, CA), mouse monoclonal antibody to cytosolic beta-hydroxybutyrate dehydrogenase (BDH2, clone 2G1, OriGene TA501293, Rockville, MD), and rabbit polyclonal antibody to acetyl-CoA acetyltransferase (ACAT1, Sigma HPA007569).

**Figure 1:**
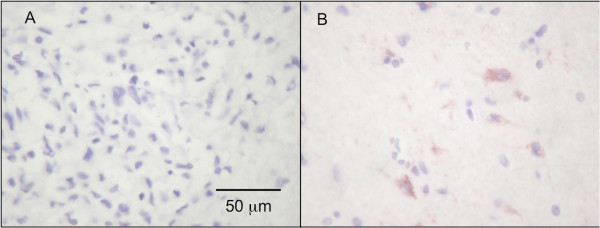
The malignant glioma cells (Case 1) show VLOW labeling for OXCT1 (A), whereas adjacent normal brain tissue show positive granular labeling pattern within neurons (B).

Immunohistochemistry reactions were scored on a semi-quantitative basis: the tumor is considered positive (POS) if greater than 20% of tumor cells are positive, LOW if 5-20% of tumor cells are positive, and very low (VLOW) if less than 5% of tumor cells are positive. Internal controls, where possible, were represented by fragments of non-neoplastic brain tissue within the same sections in which the neurons would show positive granular labeling pattern for the metabolic enzymes (Figure 1), but negative for GFAP.

## Results

**Figure 2:**
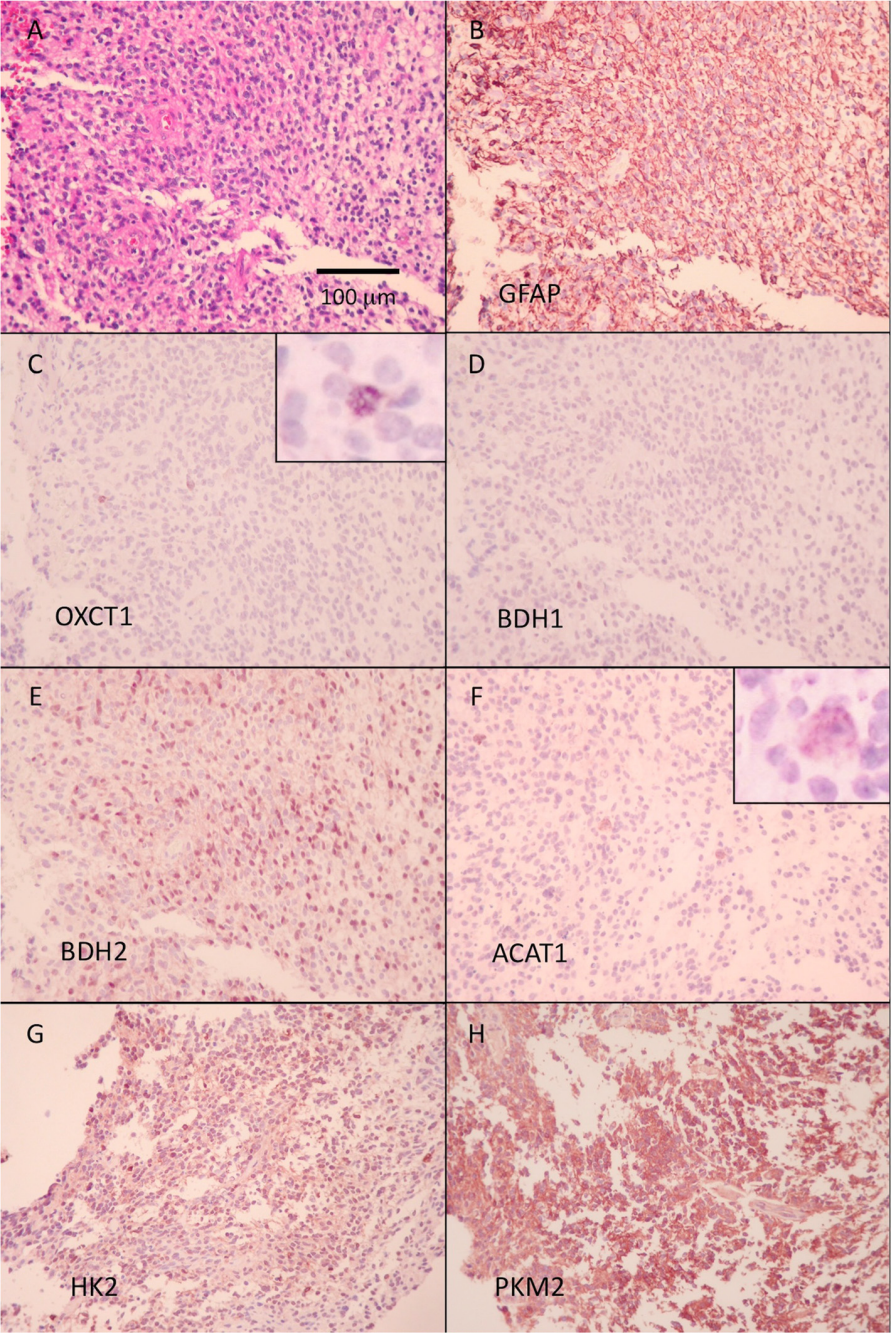
**Typical immunohistochemistry reactions of a GBM (Case #13).** H and E stained section shows a hypercellular tumor **(A)**. Immunohistochemistry reaction for GFAP **(B)** shows many positive processes in the background, but most tumor cells appear negative. This tumor is thus graded as showing LOW expression of GFAP. Stains for OXCT1 **(C)** show that most tumor cells are negative, with rare (less than 5%) cells (possibly entrapped native neurons) showing positive reaction (shown at higher magnification in the inset). Most cells also appear negative for BDH1 **(D)**, but more than 20% of cells appear positive for BDH2 **(E)**. A little more than 5% of tumor cells appear positive for ACAT1 **(F)** (shown at higher magnification in the inset). More than 20% of tumor cells are positive for the glycolytic enzymes HK2 **(G)** and PKM2 **(H)**.

Twenty two de-identified patients were studied (Table 1). The mean age was 55 with 5 females and 17 males. Each specimen was evaluated for the expression of 4 ketolytic and 2 glycolytic enzymes (Figure 2). The mitochondrial enzymes OXCT1 and BDH1 were concordantly decreased in 15 of the 22 (68%). The cytoplasmic ketolytic enzyme BDH2 along with the mitochondrial ketolytic enzyme ACAT1 were concordantly POS in 15 of the 22 (68%). Only one patient (Case #4) had all 4 ketolytic enzymes scored as LOW or VLOW.

The 2 glycolytic enzymes, HK2, and PKM2, were variably expressed in these tumors. The mitochondria-associated HK2 was often decreased (16 of 22, 72%, were either LOW or VLOW,) while the cytosolic PKM2 was more often POS (18 of 22, 81%). Of the 15 patients with concordantly decreased mitochondrial ketolytic enzymes OXCT1 and BDH1, 11 had positive expression of either one or both of their glycolytic enzymes, HK2 or PKM2. This was the most common ketolytic and glycolytic enzymatic profile observed.

## Discussion

The present results show that the ketolytic and glycolytic enzymatic profiles of malignant brain tumors were different from the normal non-neoplastic brain tissue. The most common enzymatic profile was a decrease in the mitochondrial enzymes OXCT1 and BDH1 coupled with positive expression of the glycolytic enzymes HK2 and/or PKM2. These findings support the notion that many high grade brain tumors in humans have aberrant metabolism of ketones, and may preferentially use glucose for their energy needs.

The mitochondrial ketolytic enzymes OXCT1 and BDH1 were scored as decreased (LOW or VLOW) in 15 of the 22 (68%) specimens. The mitochondria-associated glycolytic enzyme HK2 was also often decreased (16 of 22, 72%), whereas the cytosolic ketolytic (BDH2) and glycolytic (PKM2) enzymes both showed positive reactions in most of the tumors. These results suggest that many of these tumors have alterations in mitochondrial metabolism. On the other hand, the positive expression of ACAT1, also a mitochondrial enzyme, in most tumors suggests that the observed decreases of OXCT1 and BDH1 do not necessarily reflect a complete loss or absence of mitochondria enzymes in these tumors.

### Limitations of the present study

Due to limited sample size, our data cannot determine conclusively the relationships, if any, between the expression of these enzymes with respect to the tumor grades, patients’ age and gender, recent treatments or medications (e.g., steroid), other co-morbidities, or survival. Our results also cannot address the issue of whether the enzyme measurements obtained from a biopsy sample would be representative of activity in the entire tumor since the histoarchitecture of GBM can often vary significantly from one region to the next. Nevertheless, our results showing that GBMs from different patients have different expression of these enzymes are consistent with previous molecular genetic studies showing that these are genetically heterogeneous tumors [1-3]. Our results are also consistent with a recent study showing variable but positive expression of the ketone body metabolizing enzymes in several human glioma cell lines [6].

### Metabolic therapy for malignant gliomas

Recent studies in animal models have shown promising results using either non-caloric restricted KD [5,6] or calorie restriction therapy [16,17] as alternative treatment strategy for malignant gliomas. While it is well known that calorie restriction can lead to the production of ketone bodies, calorie restriction is also known to activate protective genes [4,18]. On the other hand, ketone bodies, in addition to providing alternative energy source to normal brain tissue, can also serve to activate genes that promote survival [6]. Indeed, recent studies have shown that altered tumor metabolism and epigenetic mechanisms are intimately related in the maintenance of gliomas [19].

## Conclusions

Our results suggest that the variable ketolytic and glycolytic enzyme expression profiles in malignant gliomas potentially may be useful as biomarkers to sort patients in clinical trials of KD therapy. It seems reasonable to hypothesize that patients with low or very low expression of key ketolytic enzymes (e.g., OXCT1, BDH1) in their malignant gliomas may respond better to the KD therapy than those patients with positive expression of these enzymes. Further studies in animal models and/or a large-scale clinical trial would be needed to test this hypothesis. A better understanding of the modulation of the epigenetics by metabolic enzymes in gliomas is also needed to design future therapeutic strategies.

## Abbreviations

AA: Anaplastic astrocytoma; AOA: Anaplastic oligoastrocytoma; ACAT1: Acetyl-CoA acetyltransferase 1; BDH1: 3-hydroxybutyrate dehydrogenase 1; BDH2: 3-hydroxybutyrate dehydrogenase 2; GBM: Glioblastoma; GFAP: Glial fibrillary acidic protein; GS: Gliosarcoma; HK2: Hexokinase-II; KD: ketogenic diet; LOW: low; OXCT1: Succinyl CoA: 3-oxoacid CoA transferase 1 (SCOT; EC 2.8.3.5; locus symbol OXCT); PKM2: Pyruvate kinase M2 isoform; POS: Positive; VLOW: Very low; WHO: World Health Organization.

## Competing interests

The authors declare that they have no competing interests.

## Authors’ contributions

KAS conceived of the study. KAS, LKO and HTC designed the experiments and reviewed the results. HTC wrote the first draft of the manuscript. All authors read and approved the final manuscript.
